# The psychological mechanisms of spectator experience: a pathway model of immersion, identification, and sport consumption behavior

**DOI:** 10.3389/fspor.2025.1659416

**Published:** 2025-10-29

**Authors:** Xiaogan Chen, Zhenxin Huang

**Affiliations:** ^1^ School of Physical Education, Wuxi Taihu University, Wuxi, China; ^2^ School of Health Management, Quanzhou Institute of Textile and Garment, Quanzhou, China

**Keywords:** immersive experience, team identification, sport consumption behavior, motivational regulation, psychological mechanism

## Abstract

Amid the growing convergence of experiential economy and contemporary sport contexts, the psychological mechanisms through which immersive experience drives spectator consumption remain insufficiently understood. Grounded in Self-Determination Theory, Social Identity Theory, and Flow Theory, this study proposes a structural pathway model linking immersive experience, team identification, and sport consumption behavior. Motivational regulation type (intrinsic vs. extrinsic) is introduced as a moderating variable to capture variations in the psychological transformation from affective engagement to behavioral intention. Using a questionnaire-based survey, 728 valid responses were collected and analyzed through structural equation modeling and multi-group comparison. The results reveal that immersive experience significantly enhances team identification, which mediates its influence on sport consumption. Moreover, motivational regulation plays a significant moderating role: individuals with high intrinsic motivation are more likely to follow an “immersion → identification → consumption” pathway, while those with extrinsic motivation tend to exhibit a more immediate behavioral response to immersion. These findings advance the theoretical understanding of how spectators transition from emotional engagement to consumer behavior, offering empirical support for the design of immersive sporting experiences and segmented audience management strategies.

## Introduction

Amid the rapid globalization of the sports industry and the profound restructuring of the media ecosystem, the spectator experience has become a pivotal determinant of event value and a cornerstone of sustainable industry development. Prior studies show that diverse service formats—live attendance, broadcast viewing, and sports e-commerce—enhance spectators' flow experience and strengthen team identification, thereby elevating satisfaction, loyalty, and consumption intention (1, 2). Nevertheless, this body of literature primarily examines linear relations between service delivery and outcomes, devoting insufficient attention to the psychological processes that shape spectators' cognition during sporting events. In particular, the pathways through which immersive experience translates into consumption behavior via cognitive meaning-making and motivational structures remain underexplored, with limited theoretical modeling and empirical corroboration. As a result, research on the psychology of sports consumption remains largely descriptive and fails to specify antecedent conditions or boundary mechanisms with precision.

Immersion, a central construct in experience economy theory (3), denotes a psychological state of emotional resonance and diminished boundaries with reality, elicited through heightened sensory engagement and situational absorption. In contrast to post-event evaluation indices such as satisfaction or service quality, immersion captures real-time psychological processes during sporting events. Its significance lies not only in reflecting attentional focus and affective involvement but also in demonstrating how these experiences evolve into psychological states of identification within the event context. Moreover, immersion exhibits high cross-context consistency across in-venue settings, broadcast media, and immersive technologies, thereby mitigating recall bias and extraneous attribution effects. These properties make it particularly suitable for analyzing the dynamic mechanisms underlying spectator experience.

The attentional engagement and emotional resonance elicited by immersion activate processes of identity projection that, in turn, foster team identification. This trajectory aligns with social identity theory's emphasis on belonging and value integration and resonates with self-determination theory's account of motivational internalization. Together, these perspectives provide a robust theoretical and empirical framework for explaining how spectator experiences are transformed into consumption behavior.

From a practical standpoint, immersion has emerged as a strategic priority in event operations and communication. Organizers increasingly seek to cultivate presence and affective involvement through narrative-driven atmospheres, ritualized performance settings, and the deployment of virtual reality and interactive digital technologies (4). Yet these initiatives remain insufficiently theorized and empirically validated. The psychological processes through which immersion translates into identification and subsequent consumption behavior constitute a central challenge for both sports psychology and industry research. Positioning immersion as a conceptual entry point therefore bridges the divide between practice and theory, offering a rigorous academic foundation for audience management and event design.

Whether immersion culminates in stable consumption behavior, however, is contingent upon individual motivational structures. Self-determination theory (5) posits that the degree of motivational internalization and the satisfaction of basic psychological needs determine both the depth of experiential processing and the persistence of behavioral engagement. When immersion aligns with autonomous goals, spectators are more likely to internalize emotional experiences into enduring behavioral dispositions (6, 7); conversely, immersion may remain confined to transient pleasure. Motivational regulation thus constitutes a critical threshold between immersion and identification, with divergent regulatory orientations activating distinct psychological pathways.

Building upon this theoretical foundation and addressing the identified research gap, the present study proposes an integrated structural pathway model linking immersion, team identification, and sports consumption behavior. Specifically, it investigates the mediating role of immersion in shaping consumption outcomes and incorporates motivational regulation as a moderating construct to reveal the mechanisms by which emotional perception is transformed into behavioral decision-making. By synthesizing insights from the experience economy, social identity theory, and self-determination theory, the study addresses the fundamental questions of why spectators participate, how identification is cultivated, and how these processes culminate in consumption behavior. In doing so, it advances sports-industry research beyond descriptive accounts toward an explanatory paradigm grounded in psychological mechanisms, providing both theoretical foundations and empirical avenues for the design of immersive events and spectator-engagement strategies.

## Theoretical foundations and research hypotheses

### Theoretical foundations

#### Self-determination theory and the psychological structure of sport consumption motivation

In explaining the psychological mechanisms underlying spectator decision-making in sports contexts, the structural dimensions and regulatory orientations of motivation constitute critical variables that determine behavioral stability and persistence. Self-determination theory (SDT), a leading paradigm in contemporary motivation research, posits that individuals' internalization of contextual information is contingent upon the fulfillment of three basic psychological needs—autonomy, competence, and relatedness (5). Rejecting traditional views of motivation as a unitary intensity, the theory conceptualizes motivation along a continuum from amotivation to intrinsic regulation, thereby highlighting essential differences in voluntariness and levels of internalization (8).

In sport-spectating contexts, behavioral responses to game stimuli are not driven solely by the intensity of external stimuli but are fundamentally shaped by subjective meaning-making processes. When immersive experiences satisfy spectators' needs for autonomy, provide competence-related feedback, and foster emotional connectedness, individuals are more likely to internalize such experiences as stable identity structures. Consistent with the principle of self-congruence, this internalization promotes sustained behavioral tendencies (9). Under these conditions, consumption behavior reflects intrinsic motivation, with participation arising not from external contingencies but from intrinsic drives for identity affirmation, self-expansion, and social belonging (10).

Conversely, when event contexts do not engage the psychological-needs system, or when regulation is governed predominantly by external rewards, social pressure, or utilitarian goals, behavior may still occur but tends to be unstable, short-lived, and instrumental. In such states, immersive experiences are unlikely to undergo deep processing; spectators' emotional responses and identity construction remain superficial, thereby constraining the transformative potential of sports consumption (7).

Recent scholarship has increasingly examined the predictive effects of types of motivational regulation on audience loyalty, brand relationships, and repeat-purchase intentions (11). Yet systematic frameworks remain underdeveloped regarding its role as a situational psychological mechanism that shapes the immersion–identity–behavior pathway. Motivational regulation not only determines how individuals respond to immersive stimuli but also governs whether such experiences are translated into stable identification and enduring consumption behavior. From this perspective, SDT provides a robust theoretical lens for explicating the psychological bridge between immersive experiences and behavioral responses, elucidating the intrinsic foundations of heterogeneous response patterns among spectators exposed to identical contexts. Identification emerges, and behavior endures, only when immersive experiences are perceived as congruent with spectators' psychological needs and motivational structures. Building on this foundation, further inquiry into how immersion activates cognitive–affective processing pathways leading to team identification constitutes a crucial avenue for advancing understanding of the psychological logic of sports consumption.

#### The cognitive–affective formation mechanism of immersive experience and team identification

As sports consumption becomes increasingly contextualized and immersive, spectator responses are no longer confined to rational appraisal or immediate evaluation; rather, they unfold as a holistic experiential process integrating sensory absorption, affective resonance, and identity linkage. Immersion, a central construct in experience economy theory, denotes an intensified psychological state of focused engagement and perceptual integration within a specific environment. This state allows individuals to “enter” the field of experience, temporarily dissolving boundaries between reality and mediated contexts (3). In sports spectatorship, immersion arises not only from the intensity of sensory stimulation but also from deeper psychological interactions, including emotional involvement, spatiotemporal integration, and role-based empathy. The outcome is a heightened sense of presence that engenders a perception of physical co-presence at the event (4).

Immersive experiences provide the psychological conditions necessary for cultivating team identification. According to social identity theory, individuals in social contexts seek group affiliation to fulfill needs for self-definition and value confirmation (12). Yet identification does not occur automatically; it requires a foundation of cognitive attribution and affective resonance. Within immersive states, spectators shift from “observers” to “participants” by perceiving event dynamics, simulating role behaviors, and sharing collective emotions. Through this process, the team's symbolic system becomes embedded within the individual's cognitive schema, thereby activating identification mechanisms (13).

Cognitively, immersion deepens the processing of event elements, enabling meaning construction and contextualized interpretations of team imagery, event symbolism, and value systems; these processes inform judgments of belonging (“who I am with”) and representation (“what I stand for”). Affectively, immersion activates mechanisms of collective emotional contagion, allowing individuals to experience belonging, security, and self-congruence through shared affect. These pathways are synergistic rather than independent, generating identity through a sequence of cognitive processing → affective engagement → identity internalization (14). Although prior research has advanced understanding of identity outcomes—such as loyalty behaviors, consumption preferences, and media interactions—the causal mechanisms remain theoretically fragmented, with few systematic accounts of how immersion constructs identity. Moreover, the literature focuses disproportionately on technological affordances and content presentation as sources of sensory stimulation, while underestimating contextual interpretation and psychological construction. Framing immersion as a mediating mechanism in identity formation clarifies the operational logic of spectator psychology across perceptual, affective, and symbolic dimensions and supplies the cognitive foundations and affective impetus for subsequent consumption activation.

Within the immersion–identity–behavior pathway, team identity functions as the mediating construct linking immersive experience to behavioral decision-making. It not only re-codes the psychological significance of perceptual experience but also amplifies the affective mobilization effects of event communication. Systematic modeling of this mechanism has substantial theoretical significance for explicating the psychological bases of immersive sports communication and practical utility for optimizing spectator-engagement strategies and strengthening event brand equity.

#### Flow theory and the psychological drivers of immersive experiences

Flow theory, originally proposed by Csikszentmihalyi in the 1970s, delineates an optimal experiential state in which individuals achieve a dynamic equilibrium between skill and situational challenge. In this state, they exhibit heightened concentration, temporal distortion, and a temporary dissolution of self-awareness. The theory posits that when external challenge aligns with personal capability, spectators are more likely to experience deep engagement and positive affect during sporting events, thereby generating a self-reinforcing cycle of pleasurable experience.

Within sport-spectating contexts, event tension, competitive intensity, and the ambient environment collectively constitute the challenge conditions, whereas spectators' knowledge bases, expectation structures, and community ties supply the psychological resources that support skill. When these elements align, spectators are more likely to enter a flow state that substantially enhances attentional focus and emotional absorption, thereby deepening both the intensity and the persistence of immersion (15). In this state, spectators not only derive immediate hedonic satisfaction but also experience heightened identity fusion through the interplay of positive affect and attenuated self-awareness, establishing a psychological basis for team identification (16).

In contrast to self-determination theory, which emphasizes motivational types and processes of internalization, flow theory illuminates the phenomenological characteristics of immediate psychological states, revealing the instantaneous triggers and reinforcement mechanisms of immersion. Flow simultaneously stimulates emotional engagement through the skill–challenge balance and enhances motivational drive by fostering concentrated attention and intrinsic pleasure. This mechanism explains how immersion can rapidly shape cognitive appraisals and affective responses in sporting contexts. Consequently, flow theory not only expands understanding of the psychological drivers of immersion but also provides complementary theoretical grounding for explicating its links to team identification and consumption behavior.

Taken together, immersion captures the immediate affective engagement and attentional focus that occur during sporting events; flow theory clarifies the preconditions and optimal states for experience generation; social identity theory explains how immersion translates into group belonging and identity projection; and self-determination theory underscores the decisive role of motivational internalization in sustaining behavior. Collectively, these perspectives delineate core mechanisms of spectators' cognitive processing and furnish systematic support for the pathway framework advanced in this study. Within this framework, team identification is conceptualized as the mediating construct linking immersion to consumption. Perceptual absorption and emotional responses elicited by immersion cannot yield stable, enduring consumption motives unless they are internalized through identification; identification thus constitutes the essential mechanism that transforms situational experiences into behavioral intentions. By contrast, motivational regulation does not determine whether identification occurs but shapes the strength and stability of this mechanism. According to self-determination theory, distinct regulatory orientations—intrinsic vs. extrinsic—govern whether individuals integrate immersion and identification into the self-system and extend them into persistent behavior. Motivational regulation is therefore most appropriately positioned as a moderating variable. Building on this logic, the present study specifies team identification as the mediating variable and motivational regulation as the moderating variable to clarify the transmission dynamics linking immersion, identification, and consumption, while revealing the conditional influences of individual differences on pathway effects.

### Research hypotheses

Existing research indicates that immersive experiences strengthen spectators' identity projection and group belonging by heightening attentional focus and affective engagement (13, 14). From a social identity theory perspective, immersion is not merely sensory stimulation but a psychological condition that facilitates an observer-to-member shift. In this sense, the emotional resonance and cognitive focus elicited by immersion jointly provide the affective and cognitive foundations of team identification. Accordingly, we hypothesize:

**H1.** Immersion is positively associated with team identification.

Team identification reflects more than a felt sense of group belonging; through value congruence and emotional attachment, it translates into durable behavioral tendencies. Prior studies show that highly identified spectators are more likely to return to events, purchase merchandise, and engage in sustained media interactions (1, 17). This pattern aligns with self-determination theory, which holds that internalized identification satisfies needs for autonomy and relatedness, thereby promoting persistent behavioral intentions. Thus:

**H2.** Team identification is positively associated with sports consumption behavior.

Beyond its indirect influence via identification, immersion may exert a direct effect on consumption. Kim and Ko (4) show that immersive media experiences elevate pleasure and purchase intention, and experience economy accounts similarly contend that sensory absorption and immediate affect constitute value in their own right, prompting behavior without identity mediation. Hence:

**H3.** Immersion is positively associated with sports consumption behavior.

Although immersion can directly shape consumption, evidence suggests that its more enduring influence operates through team identification (13, 14). Social identity theory emphasizes that sustained motivation requires the internalization of external experiences into identity—judgments of “who I am” and “where I belong.” Thus, identification functions as the psychological bridge linking immersion to consumption. Accordingly:

**H4.** Team identification mediates the relationship between immersion and sports consumption behavior.

Finally, the strength—rather than the mere occurrence—of the immersion → identification linkage should depend on spectators' motivational regulation. Self-determination theory suggests that intrinsically regulated individuals are more likely to integrate immersive experiences and identification into the self-system, extending involvement into long-term consumption (8, 10). By contrast, extrinsically regulated individuals rely more on rewards, sanctions, or immediate feedback, making immersion effects more transitory and affect-bound. Thus:

**H5.** Motivational regulation moderates the relationship between immersion and team identification, such that the association is stronger among intrinsically regulated spectators than among extrinsically regulated spectators.

## Research design

### Participants and sample source

This study targets young Chinese adults aged 18–35 and is designed to examine how immersive spectator experiences influence sports consumption behavior through team identification, while further investigating the moderating effect of motivational regulation within this psychological pathway. The 18–35 age range was selected because individuals in this developmental stage are consolidating social identities and consumption patterns, making them particularly susceptible to stable psychological and behavioral influences from immersive experiences. In addition, this demographic generally possesses independent financial capacity and decision-making autonomy, thereby mitigating procedural confounds associated with minors who require parental consent or proxy payments. These considerations ensure both ethical appropriateness and methodological homogeneity of the sample.

Data collection employed a combination of stratified cluster sampling and snowball sampling across seven representative cities in eastern (Shanghai, Guangzhou, Nanjing), central (Wuhan, Hefei), and western (Chengdu, Xi'an) China. The sample covered three typical audience segments: university students, young white-collar employees, and freelancers. Data were collected between March and April 2025, primarily through online distribution via the “QuestionStar” platform, supplemented by offline administration at sporting events, university clubs, and corporate activities to enhance sample diversity and representativeness. To ensure data quality, the questionnaire incorporated reverse-coded items, a “minimum viewing frequency” screening criterion, and a minimum response time of 90 s to confirm that respondents possessed genuine sports-viewing experience.

A total of 784 questionnaires were distributed, of which 728 were valid, yielding a response rate of 92.86%. Among valid responses, male respondents accounted for 53.2% and female respondents for 46.8%. The age distribution was concentrated in the 18–30 range (M = 23.41, SD = 2.96), with the 21–25 cohort representing 64.7%. Educational attainment was primarily at the bachelor's (66.8%) and master's (21.4%) levels. In terms of occupation, students comprised 54.3% and corporate employees 28.6%, with the remainder being freelancers or self-employed individuals. Screening questions at the beginning of the survey excluded respondents who reported no sports-viewing experience or who indicated they “never follow sports events,” thereby ensuring the validity of the consumption-related measures.

Furthermore, to control for potential confounding effects of individual background characteristics on the structural path analysis, gender, education level, and occupation type were incorporated as control variables in subsequent model testing. This approach enhanced both the robustness and the external validity of the analytical results.

### Measurement instruments

This study employed a structured questionnaire to collect data on core variables. All measurement instruments were drawn from internationally recognized, well-validated scales and then subjected to semantic adaptation and structural adjustment for the Chinese context. This procedure ensured theoretical alignment, content validity, and compliance with the fundamental requirements of reliability and validity for statistical analysis. The questionnaire comprised three latent variables—immersion, team identification, and sports consumption behavior—supplemented by motivational regulation as a moderating variable and demographic variables (e.g., gender, age, and viewing frequency) as controls, thereby forming a comprehensive structural path model. All items were rated on a seven-point Likert scale ranging from 1 (“strongly disagree”) to 7 (“strongly agree”).

Measurement of immersive experience was adapted from the flow dimensions proposed by Kim and Ko (4), encompassing cognitive absorption, time distortion, and enjoyment. Six preliminary items were developed to capture immersion in sport spectating. Confirmatory factor analysis (CFA) led to the elimination of three items due to insufficient factor loadings (< 0.50), collinearity, or semantic inappropriateness for spectating contexts. The removed items were: “I barely notice my surroundings while watching,” “I feel detached from reality while watching the game,” and “I feel as if I am inside the game during viewing.” The final model retained three items—“I feel completely absorbed during the game,” “Watching the game brings me pleasure,” and “My attention is highly focused during the game.” These correspond to cognitive absorption, enjoyment, and concentration, aligning with the core essence of immersion and central dimensions of flow theory. The revised model demonstrated substantially improved fit (*χ*^2^/df decreased from 4.21 to 2.87; CFI increased from 0.88 to 0.93; TLI increased from 0.85 to 0.91; RMSEA decreased from 0.092 to 0.067), indicating a robust and well-specified construct. Accordingly, this latent variable is defined as “spectator immersion,” theoretically grounded in flow and contextually relevant to sports viewing.

Team identification was assessed using the revised “Team Identification” scale by Lock, Funk, and Doyle (17), capturing cognitive, affective, and symbolic dimensions. Four items were retained, such as “I feel like I am part of this team” and “The success of this team makes me proud,” reflecting the self-affiliation–emotional identification–behavioral orientation logic of social identity theory. The scale demonstrated strong construct validity (Cronbach's *α* = 0.89, CR = 0.92, AVE = 0.76).

Sports consumption behavior was measured with reference to Trail, Anderson, and Fink's (18) dimensional framework. The initial six-item scale spanned ticket purchase, merchandise acquisition, and media engagement. Items were refined based on CFA modification criteria (factor loadings <0.50, cross-loadings, or high modification indices without theoretical justification). Two items were eliminated: “Sharing/commenting on team-related content on social media” exhibited cross-loading with team identification, reflecting identity advocacy rather than consumption intent; “Purchasing higher-priced or upgraded seats (or season tickets)” demonstrated low loadings and dependence on exogenous factors such as income, undermining unidimensionality. The retained four items represented ticket purchase/attendance, merchandise purchase, and media engagement, forming a single-factor reflective model. This structure demonstrated superior reliability and validity (Cronbach's *α* = 0.88, CR = 0.89, AVE = 0.68) compared with the original six-item version.

Motivational regulation, as a moderating variable, assessed the depth of internalization and regulatory approaches adopted in spectator experiences. The Sport Motivation Scale II [SMS-II (7);] was employed, with four subscales—intrinsic regulation, identified regulation, external regulation, and amotivation—each comprising three items, for a total of 12. Following self-determination theory (5, 19), intrinsic and identified regulation were combined into “autonomous motivation,” external regulation was treated as “controlled motivation,” and amotivation remained independent. This categorization aligns with the SMS-II developmental framework and subsequent validation studies (7), avoiding distortions caused by rigid binary classifications. Subscale scores were computed as item means, standardized, and aggregated into three indicators—autonomous motivation, controlled motivation, and amotivation—for multigroup analyses and moderation tests. All subscales demonstrated robust reliability and validity, supporting their use in subsequent path analyses.

The draft questionnaire underwent three rounds of expert review and linguistic refinement by sports psychology specialists to ensure clarity and semantic precision. A pilot test (*N* = 30) confirmed structural coherence and logical consistency. CFA on the full sample (*N* = 728) verified satisfactory psychometric properties: composite reliability (CR) exceeded 0.80 and average variance extracted (AVE) exceeded 0.60 for all latent constructs. Model fit indices also demonstrated strong performance (*χ*^2^/df = 2.45, CFI = 0.962, TLI = 0.951, RMSEA = 0.045, SRMR = 0.032), confirming sound construct validity and reliable measurement for subsequent structural analyses.

### Data analysis methods

Data organization and statistical analysis were conducted using SPSS 27.0 and AMOS 26.0. The overall procedure comprised three stages: (1) descriptive statistics of sample characteristics and inter-variable relationships, (2) validation of the latent-variable measurement model, and (3) construction and testing of the structural path model.

First, descriptive analyses in SPSS examined demographic characteristics of the valid sample, including gender, age, education level, occupation type, and viewing frequency. Means, standard deviations, and ranges for each latent variable were calculated to assess central tendency and dispersion. Pearson correlation analysis was then employed to evaluate linear associations among immersion, team identification, sports consumption behavior, and motivational regulation. Data normality was assessed using skewness and kurtosis indices, while multicollinearity and outlier diagnostics were conducted to mitigate the influence of anomalous cases on model estimation.

Second, the latent-variable measurement model was tested using confirmatory factor analysis (CFA). Standardized factor loadings were estimated with maximum likelihood (ML) procedures. Composite reliability (CR), average variance extracted (AVE), and Cronbach's *α* were computed to assess internal consistency, convergent validity, and reliability of latent variables. Model fit was evaluated with multiple indices, including *χ*^2^/df, Comparative Fit Index (CFI), Tucker–Lewis Index (TLI), and Root Mean Square Error of Approximation (RMSEA), to ensure the adequacy and validity of the measurement structure.

Based on these results, a structural equation model (SEM) was specified, with immersion as the independent variable, sports consumption behavior as the dependent variable, team identification as the mediating variable, and motivational regulation as the moderating variable. The model was designed to test both the significance and robustness of the hypothesized relationships. Mediation effects were examined using bias-corrected bootstrap resampling (5,000 iterations). Confidence intervals were employed to evaluate the statistical significance of indirect effects, thereby avoiding the distributional assumptions inherent in Sobel tests and enhancing the robustness of mediation analysis. To control for heterogeneity, gender, age, education level, occupation type, and viewing frequency were included as control variables, thereby strengthening the internal validity and explanatory power of the model.

Finally, multi-group SEM was conducted to assess moderation effects across motivational subgroups. Following established procedures (25), configural, metric, and intercept invariance were sequentially tested to establish measurement equivalence. After confirming at least metric invariance, structural invariance was assessed by constraining key path coefficients to equality across groups and comparing the fit of constrained and unconstrained models. Group-level differences were evaluated using chi-square difference tests (*Δχ*^2^) and changes in incremental fit indices (*Δ*CFI, *Δ*TLI). A *Δ*CFI or *Δ*TLI greater than 0.010, or a statistically significant *Δχ*^2^, indicated meaningful cross-group differences in structural path effects (26). This procedure ensured rigorous testing of group-level moderation while maintaining measurement invariance, thereby providing robust validation of the hypothesized moderating effects.

## Research results

### Sample characteristics and variable distributions

To elucidate the structure of spectators' psychological experience during sporting events, statistical tests were conducted on three core variables—immersion, team identification, and sports consumption behavior (see Table 1). Immersion showed a relatively high mean (M = 5.08, SD = 0.87), indicating that respondents generally exhibited strong attentional focus and emotional engagement during competitions. By contrast, team identification (M = 3.13, SD = 0.98) and sports consumption behavior (M = 3.11, SD = 0.93) displayed lower means, evidencing a progressive attenuation from psychological involvement to identity formation and, ultimately, behavioral intention. This disparity suggests that, although immersive experiences are commonly experienced, their extension to social identification and consumption behavior depends on specific contextual conditions.

**Table 1:** Descriptive statistics of core variables (N = 728).

Variable	M	SD	Min	Max
Immersion	5.08	0.87	2.18	7.00
Team Identification	3.13	0.98	1.00	6.18
Sport Consumption	3.11	0.93	1.00	7.00

All items measured on a 7-point Likert scale.

M, mean; SD, standard deviation.

Correlation analyses (Table 2) revealed significant positive relationships among all three variables (*p* < .001). Immersion and team identification were moderately correlated (*r* = 0.60), whereas the strongest association occurred between team identification and sports consumption behavior (*r* = 0.65), delineating a continuous pathway from emotional investment to behavioral orientation. Although immersion and sports consumption behavior were also significantly associated (*r* = 0.55), the comparatively weaker coefficient suggests that behavioral responses rely more heavily on the mediating role of team identification.

**Table 2:** Pearson correlation matrix among core variables (N = 728).

Variable	Immersion	Team identification	Sport consumption
Immersion	1.00	0.60***	0.55***
Team Identification	0.60***	1.00	0.65***
Sport Consumption	0.55***	0.65***	1.00

Values are Pearson correlation coefficients.

*** *p* < .001.

**Figure 1:**
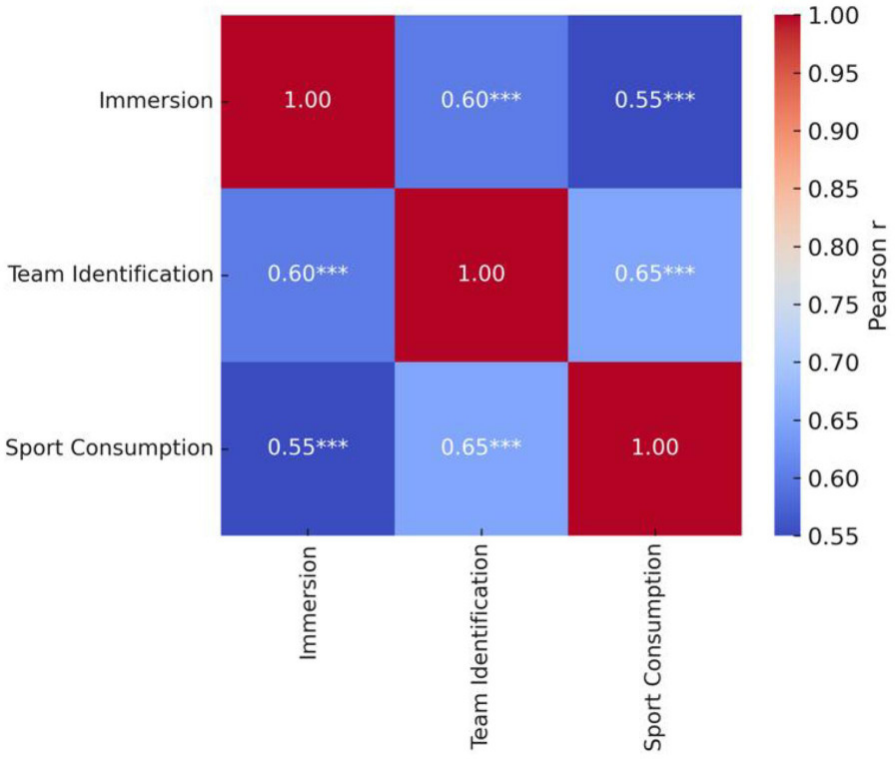
Heatmap of correlations among core variables. Pearson correlation coefficients are shown. ***p < .001.

Figure 1 visualizes these associations via a heat map. No directional inconsistencies or structural discontinuities were detected among the three variables; the overall correlation pattern was compact and stable. The consistency of significant associations further supports the proposed path structure and its theoretical validity.

In sum, spectators' immersive experiences during sporting events channel sustained attention and consumption-oriented responses through the development of team identification. These results provide a sound foundation for subsequent analyses of mediating pathways and moderating mechanisms within structural models.

### Validation of the latent variable measurement model

To ensure the statistical validity and theoretical soundness of the latent structural model, the measurement model was rigorously examined prior to structural path analysis. This procedure aimed to confirm the measurement quality and structural adequacy of three core latent variables—immersion, team identification, and sports consumption. Measurement dimensions for each construct were derived from established theoretical frameworks and refined through multiple rounds of pretesting. Specifically, immersion was conceptualized as a two-dimensional structure comprising cognitive absorption and affective engagement; team identification was defined by sense of belonging and emotional bonding; and sports consumption encompassed behavioral intention and willingness to pay.

Table 3 reports the number of items, means, standard deviations, and internal-consistency coefficients for the three latent variables. Results indicate that immersion (*α* = 0.91), team identification (*α* = 0.89), and sports consumption (*α* = 0.88) each exhibit high internal consistency, substantially exceeding the conventional benchmark of 0.70. These findings confirm that the instruments possess strong structural stability and effectively capture respondents' subjective psychological experiences.

**Table 3:** Latent constructs and reliability.

Construct	No. of Items	Mean	SD	Cronbach's *α*
Immersion	3	5.08	0.87	0.91
Team Identification	4	3.13	0.98	0.89
Sport Consumption	4	3.11	0.93	0.88

α, Cronbach’s alpha; SD, standard deviation.

To further evaluate factor structure and fit, confirmatory factor analysis (CFA) was conducted, with results summarized in Table 4. The overall model fit was satisfactory: *χ*^2^/df = 2.45, meeting the empirical benchmark (<3) commonly applied in structural modeling. Incremental fit indices were strong (CFI = 0.962; TLI = 0.951), indicating high explanatory power and parameter stability. Error indices were within recommended thresholds (RMSEA = 0.045 < 0.06; SRMR = 0.032 < 0.08), suggesting minimal residual misspecification and close alignment between the model structure and observed data.

**Table 4:** Model fit indices for the measurement model.

Fit index	Value	Recommended threshold
*χ*^2^/df	2.45	<3
CFI	0.962	>0.95
TLI	0.951	>0.95
RMSEA	0.045	<0.06
SRMR	0.032	<0.08

CFI, comparative fit index; TLI, Tucker–Lewis index; RMSEA, root mean square error of approximation; SRMR standardized root mean square residual.

Collectively, these results demonstrate close convergence between theoretical assumptions and empirical evidence, supported by robust reliability and construct validity. This validation provides a solid foundation for subsequent structural path analysis and the exploration of moderating mechanisms.

### Structural path model Fit and hypothesis testing

After confirming the reliability and validity of the measurement model, a structural path model was constructed to examine the causal relationships among immersion, team identification, and sports consumption behavior. Model fit indices are reported in Table 5: *χ*^2^/df = 2.31, CFI = 0.957, TLI = 0.945, RMSEA = 0.047, and SRMR = 0.038, indicating satisfactory overall fit. Although the TLI falls marginally below the ideal threshold of 0.95, it remains within commonly accepted ranges, suggesting that the model adequately represents the observed data and exhibits robust explanatory power.

**Table 5:** Structural model fit indices.

Fit index	Value	Recommended threshold
*χ*^2^/df	2.31	<3
CFI	0.957	>0.95
TLI	0.945	>0.95
RMSEA	0.047	<0.06
SRMR	0.038	<0.08

Model fit was evaluated against conventional SEM criteria.

CFI, comparative fit index; TLI, Tucker–Lewis index; RMSEA, root mean square error of approximation; SRMR, standardized root mean square residual; *χ*^2^/df, Chi-square divided by degrees of freedom.

**Figure 2:**
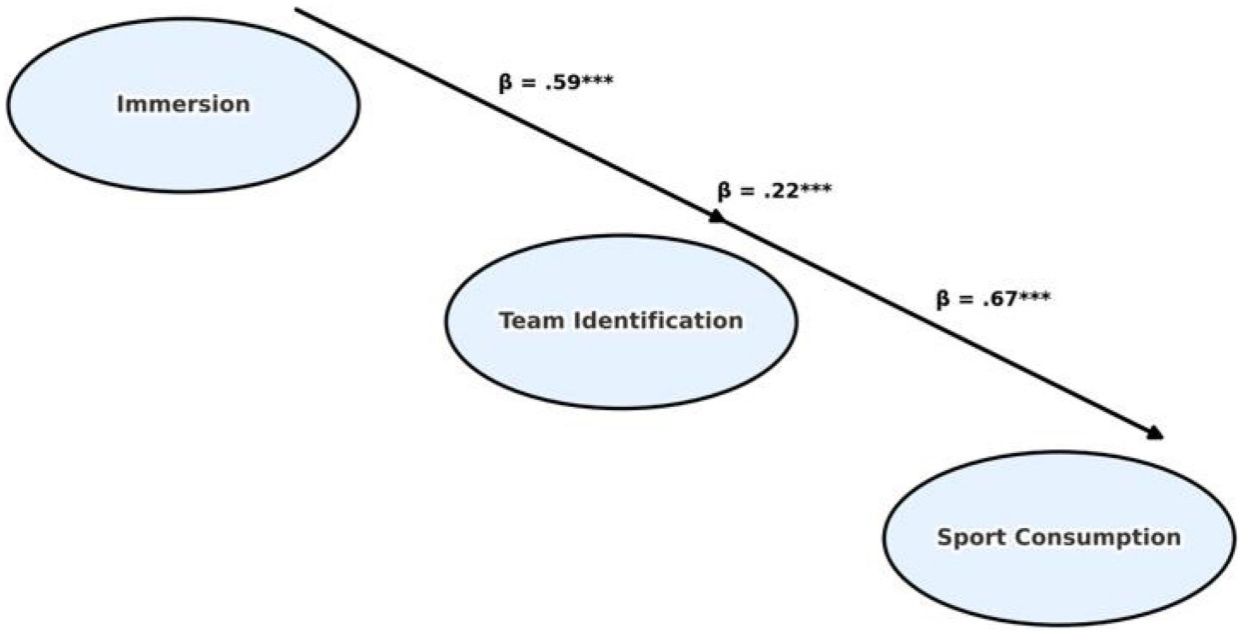
Structural model with standardized path coefficients.

Path estimates are presented in Table 6 and Figure 2. Immersion exerted a significant direct effect on sports consumption behavior (*β* = 0.22, *p* < .001), demonstrating that heightened spectator immersion can drive consumption tendencies even in the absence of mediation. Moreover, immersion produced a substantial indirect effect through team identification: the standardized coefficient from immersion to team identification was 0.59 (*p* < .001), and from team identification to sports consumption behavior was 0.67 (*p* < .001). These results indicate that the psychological attachment fostered by immersive experiences strengthens individuals' emotional bonds with their supported teams, which in turn translates into stronger consumption motivation.

**Table 6:** Standardized path coefficients and significance.

Pathway	*β* (Standardized)	SE	CR	*p*-value	Significance
Immersion → Team Identification	0.59	0.04	14.75	<.001	***
Team Identification → Sport Consumption	0.67	0.05	13.40	<.001	***
Immersion → Sport Consumption	0.22	0.03	7.33	<.001	***

*β*, standardized regression coefficient; SE, standard error; CR, critical ratio.

*** *p* < .001.

A comparison of coefficient magnitudes shows that, relative to immersion's direct effect (*β* = 0.22), its indirect effect via team identification (0.59 × 0.67 ≈ 0.40) is considerably stronger. This pattern highlights the predominance of the “immersion → identification → consumption” pathway. The evidence not only supports the three hypothesized relationships but also underscores the central role of mediation in the transition from experiential states to behavioral outcomes.

From a theoretical standpoint, these findings validate the proposed model in which situational immersion activates social identification mechanisms that, in turn, drive behavioral intent. This mechanism aligns with self-determination theory's emphasis on the fulfillment of relational needs in motivating behavior, showing that immersive experiences extend beyond immediate affective gratification to channel individuals toward consumption-oriented actions by reinforcing identity recognition and collective belonging.

In summary, the structural path model delineates a clear psychological chain linking immersion, team identification, and sports consumption behavior. The mediating role of team identification functions as a critical bridge, underscoring the foundational significance of identity mobilization within immersive sports communication environments.

### Moderation analysis: multi-group comparison based on types of motivational regulation

To further examine the moderating effect of motivational regulation types on structural path relationships, a multi-group structural equation modeling (SEM) approach was employed. Participants' motivational regulation levels (intrinsic regulation vs. extrinsic regulation) served as grouping variables to investigate whether significant differences existed in the coefficients of three structural paths across the two subgroups. Prior to conducting formal cross-group path comparisons, the measurement invariance of the model was first tested. Results indicated no significant difference between the configuration invariance model and the free model [*Δχ*^2^(df = 12) = 18.42, *p* = .103; *Δ*CFI = 0.004], indicating consistent latent factor structures across groups. Further constraints on factor loadings revealed no significant difference in model fit between the measurement invariance model and the configuration model [*Δχ*^2^(df = 10) = 12.60, *p* = .246; *Δ*CFI = 0.006]. Therefore, the model can be considered to achieve at least measurement invariance in cross-group comparisons, providing a prerequisite for subsequent structural path comparisons. Further structural invariance testing revealed significant differences in the “Immersion → Identification” path between the intrinsic regulation group and the extrinsic regulation group [*Δχ*^2^(df = 1) = 4.87, *p* = .027], and the “identification → consumption” path also differed significantly [*Δχ*^2^(df = 1) = 5.93, *p* = .015]. Conversely, the “immersion → consumption” path was significantly stronger in the external regulation group than in the internal regulation group [*Δχ*^2^(df = 1) = 7.42, *p* = .006]. Table 7 presents the standardized estimates (*β*), standard errors (SE), and *χ*^2^ difference tests for each path across the different moderation groups.

**Table 7:** Standardized path coefficients by motivation regulation type (multi-group SEM).

Structural Path	*β* (Intrinsic)	SE	*β* (Extrinsic)	SE	*Δχ*^2^ (df = 1)	*p*-value
Immersion → Team ID	0.63	.04	0.52	.05	4.87	.027
Team ID → Consumption	0.72	.05	0.61	.06	5.93	.015
Immersion → Consumption	0.15	.03	0.31	.04	7.42	.006

Standardized path estimates are reported for intrinsic and extrinsic motivation groups. Δχ^2^ values represent the chi-square difference test between constrained and unconstrained models (df = 1). p < .05, p < .01.

Results indicate that the path coefficient for immersion on team identification was 0.63 (SE = .04) in the intrinsic regulation group and 0.52 (SE = .05) in the extrinsic regulation group, with a significant difference between the two (*Δχ*^2^ = 4.87, *p* = .027). This suggests that the construction of social belonging through immersive experiences is more pronounced among individuals with high intrinsic motivation. Team identification's influence on sports consumption behavior was also significant in both groups. However, in the intrinsic regulation group (*β* = 0.72, SE = .05) than in the extrinsic regulation group (*β* = 0.61, SE = .06), with the difference also reaching statistical significance (*Δχ*^2^ = 5.93, *p* = .015). This suggests that the behavioral conversion pathway triggered by team belonging holds greater motivational potential among individuals with stronger self-regulation.

In contrast to the above path, the direct path from immersion to sports consumption exhibited the same direction in both groups. However, the path coefficient was higher in the extrinsic regulation group (*β* = 0.31) and significantly superior to that in the intrinsic group (*β* = 0.15), with this difference also being statistically significant (*Δχ*^2^ = 7.42, *p* = .006). This finding reveals that for individuals reliant on external motivators, immersive experiences are more readily converted into consumption behavior directly, bypassing the motivational transformation mediated by team belonging.

**Figure 3:**
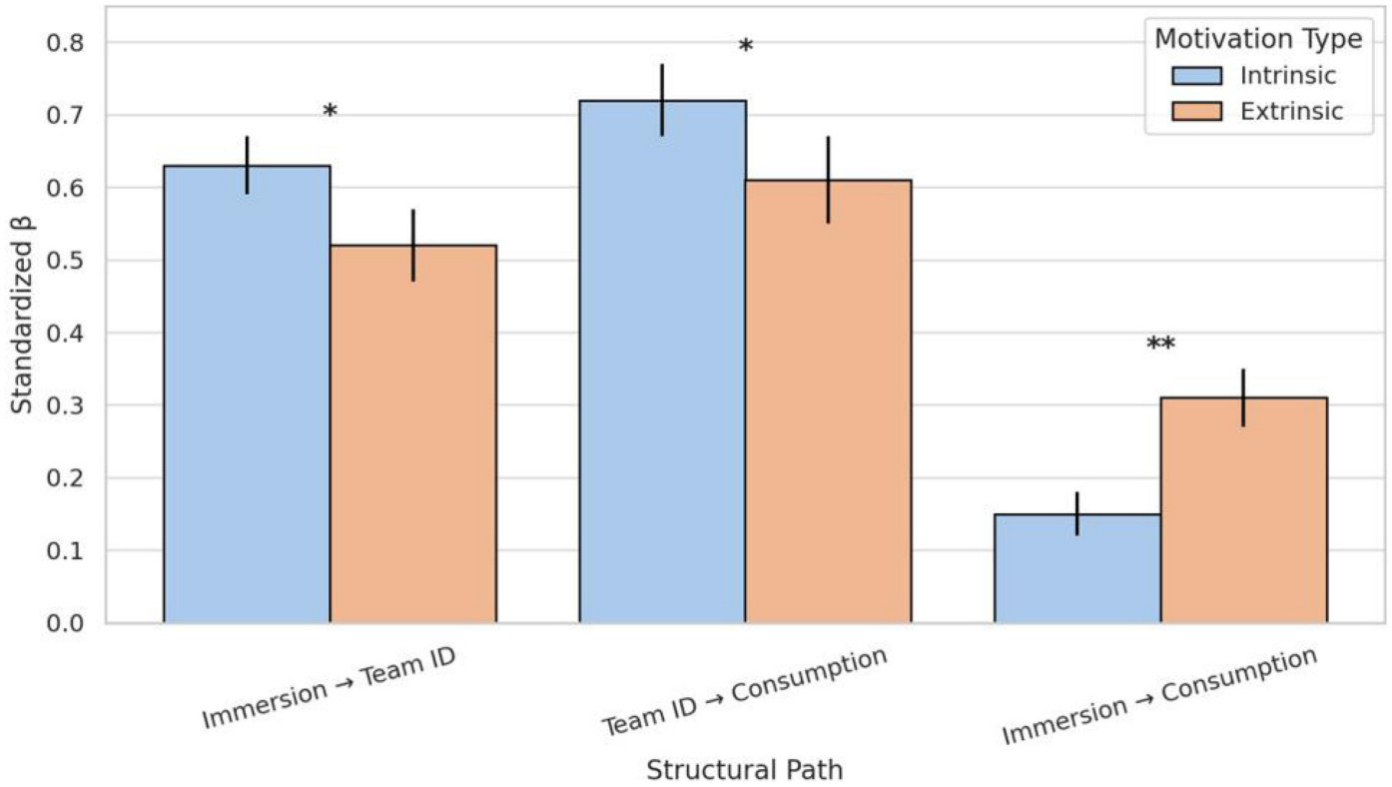
Standardized path coefficients by motivation regulation type. Bars indicate standardized *β* values for each structural path across intrinsic and extrinsic motivation groups. Error bars represent standard errors. *p* < .05, *p* < .01 (between-group difference).

Figure 3 further visualizes the cross-group differences in path coefficients, illustrating comparative characteristics of estimated values and significance levels across the three primary pathways under varying motivational regulation levels. Overall, the moderating effect of motivational regulation type on structural paths is significant, revealing distinct psychological mechanisms: intrinsically regulated individuals rely more on identity mechanisms to activate behavior, while extrinsically regulated individuals respond more directly to the stimuli inherent in the immersive experience itself.

In summary, motivational regulation type exerts a significant moderating effect on the structural paths linking immersion, team identity, and sports consumption. Specifically, intrinsically regulated individuals rely more heavily on team identification to stimulate consumption behavior, exhibiting a stronger indirect effect pattern. In contrast, extrinsically regulated individuals are more directly driven by the immersive experience itself, exhibiting a more reactive consumption tendency. These differences indicate that audiences with different regulation types exhibit significant divergence in information processing and motivational construction mechanisms, necessitating their identification and differentiation in practical communication strategies.

## Discussion

### From immersive experience to consumption intention: a progressive construction of psychological mechanisms

Research indicates that although immersive experiences generally elicit high audience engagement, their influence on sports consumption behavior is not direct; rather, it requires a psychological intermediary—team identification—to translate experience into behavior. This pattern suggests that, while immersion provides intense perceptual involvement and affective mobilization, its behavioral guidance remains latent without internalized identification with a team's symbolic system. An individual's situational absorption does not automatically trigger behavior; it depends on a psychological transformation from sensory experience to identity affiliation. From a flow theory perspective, immersion is more likely to escalate into a flow state—and thereby acquire behavioral translation potential—when the event context achieves a challenge–skill balance, supported by clear goals and immediate feedback (20).

As a core construct in experience economy theory, immersion entails perceptual fusion with the situation and affective engagement (3). Within spectator-sport contexts, immersion intensifies psychological alignment with the event space, generating a subjective sense of presence. Through mechanisms such as attentional focus, dissolution of reality boundaries, and role-based empathy, it fosters deep experiential engagement (4). However, whether cognition and affect activated by immersion shape behavior depends on whether a path of social identification is triggered. If immersion does not translate into identification with team symbols, a sense of belonging to collective emotions, and self-positioning within shared meanings, its impact on consumption behavior is unlikely to persist. This mechanism accords with the hierarchical logic reported by audience experience scales: situational absorption must first be anchored in recognizable team symbols and community cues before entering behavior-oriented decision pathways (2).

Path analyses further corroborate the sequential nature of this psychological transition. Although immersion directly affects sports consumption behavior, its indirect pathway via team identification is larger and has greater explanatory power, underscoring the mediating force of identification within the decision chain. This pattern aligns with Lock and Heere's (13) identity-generation model, whereby immersive experiences activate social belonging to teams, transforming sporting events from external occurrences into integral components of personal identity systems. In this sense, sports consumption behavior is not a transient reaction but an externalized enactment of identity affirmation and value expression. In line with Lock et al. (17), when identity structures are stable, the marginal effects of immersion more often manifest in relationship deepening and loyalty, forming an identity-driven route to sustained consumption. Consistent with flow research, optimal experiences yield durable aftereffects—“high-quality memories” and “willingness to re-participate”—that consolidate identification-mediated influence in the post-event phase, helping explain the greater robustness of indirect effects (20).

From the standpoint of motivational internalization, self-determination theory posits that external experiences become internal behavioral tendencies only when psychological needs are satisfied and identity structures are supported (5). Immersive experiences alone seldom translate directly into consumption because they lack sufficient motivational structure. Through identification, however, individuals integrate group symbols formed during events with personal value systems via self-consistency pathways, thereby generating spontaneous and sustained intentions (9). Thus, immersion functions as a motivational precursor rather than a terminal driver; its psychological efficacy is realized within an identity framework. This interpretation aligns with evidence on differentiated consumption motives: when self-regulation is high, audiences prefer identity-mediated pathways over short-term stimuli to convert experience into action (7).

In summary, the three-stage chain immersion–identification–behavior reveals a nonlinear architecture of spectators' psychological responses. Immersion supplies a perceptual route to motivation, whereas team identification constitutes the central driver of action. Consequently, designing immersive sports content requires more than technical sensory stimulation; it must be complemented by value narratives, identity construction, and collective belonging to forge a viable pathway from sensory engagement to social mobilization.

### The mediating role of team identification: activation mechanisms and pathway interpretation

Team identification, as a key mediating variable between immersive experiences and sports consumption behavior, functions not merely as a statistical connector but as a mechanism that internalizes immersive states at the sociopsychological level and transforms them into behavioral motivation. Empirical findings indicate that, relative to the direct influence of immersion on sports consumption behavior, the indirect pathway through team identification exhibits greater explanatory power and predictive utility. This structural distinction suggests that the behavioral significance of immersion is not immediate; it requires cognitive–emotional transformation via identification mechanisms to achieve meaning construction and motivational integration. Within this process, event rhythm, goal cues, and on-site feedback collectively shape a sequential processing chain—“attention fixation → enhanced sense of control → meaning extraction”—that approaches optimal processing efficiency and increases the likelihood of encoding and retrieving social cues.

The mediating role of team identification derives from the integration of three psychological functions: cognitive affiliation, affective valuation, and behavioral orientation. Social identity theory posits that individuals construct self-concept through group membership, thereby satisfying fundamental needs for belonging, security, and self-consistency (12). In immersive spectator environments, audiences progressively form symbolic connections with supported teams through event participation, emotional contagion, and value resonance. This transformation redefines the spectator's role from “observer” to “member,” rendering the event not merely an external setting but an integral component of the self-system (21). When chants, rhythms, and emotional fluctuations in the stands synchronize, individuals experience “coordinated convergence” in temporal perception and behavioral intent. Such shared experiences amplify the salience of collective goals and team symbols, reduce uncertainty in identity judgments, and reinforce the stability of identification.

In this study, immersion primarily activated team identification by functioning as a situational catalyst. Immersion serves not only as attentional absorption but also as a mechanism for intensifying emotional resonance. Under high-tension scenarios and collective emotional contagion, spectators are more likely to internalize a team's symbolic system into their cognitive structures and affective baselines. This process reflects Madrigal's (14) conceptualization of “the shift from experiential involvement to identity projection,” whereby audiences confirm social belonging through emotional resonance and subsequently reconstruct self-boundaries. When immersion aligns with salient team symbols and community cues, identification becomes more firmly consolidated, evolving into loyalty and sustained participation (22). Conversely, when feedback is ambiguous, rhythms are misaligned, or perceptions of control are attenuated, immersion may temporarily heighten emotion yet fail to yield meaningful integration, leaving identification in a fragile state that cannot sustain subsequent motivation.

Importantly, team identification is not a passive intermediate outcome of immersion but an actively generated psychosocial construct. Activating identification requires complex processing, including meaning construction, emotional bonding, and role internalization. Its formation depends on the depth of immersion, the symbolic salience of the event context, and individuals' sensitivity to social needs. Consequently, this mediating pathway is not universal; it manifests most strongly in specific cultural settings, high-engagement scenarios, and audience segments characterized by pronounced identity overlap (23). In such cases, team symbols often intertwine with regional, historical, or community narratives, lending stability to identification and sustaining consumption intentions and participation intensity (21).

In summary, team identification plays an indispensable mediating role in the immersion–behavior pathway. Its function extends beyond serving as a conduit; it constitutes the intrinsic mechanism through which immersive experiences acquire behavioral meaning. By activating identity structures, audiences integrate emotional resonance, role internalization, and value acceptance into a coherent system, thereby generating stable behavioral tendencies. The identification mechanism elevates immersion from a sensory state to a social motive, endowing sports consumption with the dual significance of identity expression and collective belonging.

### Divergent effects of motivational regulation: a psychological reconstruction of moderation mechanisms

Multi-group structural path analysis demonstrates that motivational regulation exerts a significant moderating effect on the overall relationships within the immersion → team identification → sports consumption behavior pathway. Specifically, intrinsically regulated individuals exhibit higher standardized coefficients on both the immersion → identification and identification → consumption paths than extrinsically regulated individuals. Conversely, on the direct immersion → consumption path, extrinsically regulated individuals display greater response magnitude. These results suggest that motivational structures not only influence the strength of behavioral responses to external stimuli but also shape fundamental patterns of information processing and pathway selection. Under dominant intrinsic regulation, the coupling between task goals and subjective value facilitates sustained attentional focus and enhanced action control. This state approximates optimal experience, thereby improving the efficiency of meaning extraction and integration from team-related cues.

According to self-determination theory, the degree of motivational internalization determines how individuals process meaningful information in external contexts. Intrinsically regulated individuals integrate external experiences into the self-system, demonstrating greater autonomy and goal consistency (5). In immersive spectator settings, they more readily incorporate symbolic systems, team values, and collective emotions into self-construction, thereby fostering psychological belonging and team identification. Consequently, for such individuals, immersive experiences constitute not merely sensory engagement but also preconditions for identity activation, with behavioral responses primarily realized through social identification (10). This explains why the immersion → identification → consumption pathway yields stronger indirect effects among intrinsically regulated groups. In live spectator contexts, continuity of focus, compressed time perception, and heightened action control often co-occur; although this state does not directly drive behavior, it substantially enhances meaning integration during identity formation, solidifying the transition from emotional arousal to identity positioning.

By contrast, the behavior of extrinsically regulated individuals is driven primarily by reward–punishment contingencies, social evaluation, or instrumental goals. Their motivational structure relies less on internal consistency, rendering them more inclined to respond directly to situational stimuli (8). During immersive experiences, such individuals exhibit strong sensory absorption but lack the motivational foundation necessary for robust identity formation. As a result, they may develop preliminary consumption intentions without relying on identification mechanisms. In this pathway, immersion functions more as an immediate incentive, with behavioral drive reactive rather than internalized. Consequently, responses are more vulnerable to environmental fluctuations, producing greater behavioral volatility.

Intergroup differences align with prior findings in sports-consumption research. Pelletier et al. (7) showed that individuals with higher intrinsic motivation exhibit greater consumption loyalty and behavioral consistency, forming durable brand attachments. In contrast, externally regulated individuals are more susceptible to transient influences such as affect, promotions, or peer communication; their consumption intentions lack identity-based stability and thus exhibit higher uncertainty. The present study replicates and extends these insights, reinforcing the critical moderating role of motivational regulation in shaping consumption pathways. Moreover, motivational effects are not uniform: they vary across cultural settings, event types, and community contexts. Cross-cultural research indicates that in collectivist cultures, self-identity is constructed through social belonging, making intrinsic regulation more effective in reinforcing identification pathways, whereas in individualist contexts, extrinsic incentives more directly stimulate consumption (24). Event significance and intensity also matter: in landmark or high-tension competitions, even extrinsically regulated individuals may experience temporary identification due to the atmosphere, though such effects lack long-term stability. Community interaction likewise plays a pivotal role; cohesive fan communities can foster gradual internalization among externally regulated individuals through sustained communication and value reinforcement, thereby stabilizing identity structures (1).

From a mechanistic perspective, motivational regulation does not alter the existence of path relationships but rather the psychological foundation on which they operate. Intrinsic regulation provides the endogenous condition for the identification mechanism, enabling immersion to trigger genuine constructions of social belonging. Extrinsic regulation, by contrast, depends more on stimulus intensity and immediate feedback. Although the path structure may appear similar, its psychological processes remain more transient, instrumental, and unstable. This divergence underscores that individuals do not respond uniformly to identical immersive experiences. Structural models therefore reveal differentiated psychological foundations under distinct motivational orientations and should not be applied mechanically. Accordingly, enhancing goal clarity, optimizing the perceptibility and controllability of on-site feedback, and encouraging reflective engagement with value narratives before and after events are more effective than merely intensifying promotional stimuli for strengthening meaning processing and stabilizing behavioral responses across motivational types.

It is also important to note that this study includes both live spectators and media viewers. Although the psychological essence of immersion is shared, the environmental drivers differ. Live spectators rely more on collective atmosphere, audience interaction, and ritualized contexts, whereas media viewers are more influenced by broadcast quality, commentator engagement, and digital interactivity. This distinction implies that the immersion → identification → consumption pathway requires context-specific interpretation. For live audiences, optimizing stadium ambiance and collective interaction is essential; for media viewers, improving broadcast technology and narrative engagement is critical. Future research could employ multiple SEM models or hierarchical analysis to compare pathway strength across these groups, thereby informing targeted strategies for sports marketing and consumption promotion under diverse service models.

In summary, motivational regulation significantly moderates the immersion → identification → consumption pathway, reflecting individual differences in information processing, social belonging, and behavioral drive. Intrinsically regulated individuals rely on identification mechanisms to integrate emotional experiences into the self-system, thereby generating stable consumption motivation. In contrast, extrinsically regulated individuals respond more directly to immersive stimuli, producing stronger but less stable consumption intentions.

### Structural features of spectator psychology and theoretical mapping

Immersive experience, team identification, and motivational regulation are not independent variables but interdependent mechanisms nested within one another. Together, they sequentially shape spectators' behavioral decision-making, forming a structured psychological processing system. Immersive experience provides the perceptual and affective conditions for absorption and situational focus. However, if it fails to activate social identification, the emotions it evokes lack durability and cannot serve as enduring sources of behavioral motivation. Without entering the pathway of identity construction, emotional mobilization remains confined to the experiential level. Team identification bridges immersion and action by integrating symbolic recognition and collective emotional bonding, thereby transforming situational engagement into self-positioning. Yet this mechanism is not automatic; it depends on whether spectators possess an internally integrated motivational structure. While identification may provide a conduit for perceiving social meaning, it translates into consumption intention only when conditions for motivational internalization are satisfied. Among audiences predominantly characterized by external regulation, identity—even if formed—often remains superficial, lacking the sustained force necessary to guide behavior.

Theoretically, the pathway mechanism reflected in this model exhibits convergent logic across several paradigms. Flow theory specifies the antecedent conditions and processing dynamics for optimal experience within the immersion module, showing that challenge–skill balance, clear goals, and immediate feedback enhance attentional continuity, sense of control, and meaning extraction. Within the experience-economy framework, immersion represents a perceptual–affective mechanism. Team identification is rooted in social identity theory's interpretation of belonging, while motivational regulation reflects self-determination theory's account of conditions for generating and sustaining motivation. Collectively, these frameworks form complementary psychological pathways for behavioral generation: situational triggers rely on immersion, social meaning depends on identity construction, and motivational fulfillment requires internalization within the intrinsic motivation system. The flow mechanism acts as an “efficiency amplifier,” facilitating the encoding of immersion-derived information into retrievable social cues while reducing uncertainty in identity judgments. Emotional responses, identity formation, and motivational tendencies operate sequentially within this structural pathway, constituting the psychological foundation for transforming spectator experiences into sports consumption. When flow antecedents are present, this linkage becomes more stable and motivational representations become more enduring; in their absence, responses remain transient and affect-driven. Within this mechanism, emotion functions as the entry point, identity as the bridge, and motivation as the endpoint—all indispensable to the process.

In summary, immersive experience, team identification, and motivational regulation constitute three interlocking psychological mechanisms underlying spectator behavior. They operate in progressive stages: situational processing, identity construction, and motivational generation. Immersion supplies perceptual and affective conditions; identification conveys social meaning and secures identity positioning; and motivational regulation determines the organization of behavioral motives and the stability of responses. Their synergistic interaction constructs a nested psychological system, explaining why spectators exhibit systematic differentiation in pathway selection when confronted with similar circumstances.

## Research conclusions and recommendations

### Conclusions

This study constructs and validates a structural path model of Immersion–Team Identification–Sports Consumption Behavior, grounded in self-determination theory, flow theory, social identity theory, and the experience economy framework. It systematically elucidates the psychological mechanisms and behavioral logic underlying spectators' responses in immersive viewing contexts. The main conclusions are as follows:

(1) Immersive experiences significantly enhance team identification. Under conditions of heightened immersion, spectators readily develop psychological belonging and identity projection toward their supported teams. This pathway exerts a consistently positive effect, demonstrating that immersion provides both the emotional foundation and the cognitive prerequisite for activating identification mechanisms.
(2) Team identification strongly predicts sports consumption behavior. Individuals with higher levels of identification display greater willingness to purchase tickets, acquire merchandise, and engage in event-related promotion. Thus, identification operates as a pivotal bridge that translates emotional arousal into behavioral intention.
(3) Immersive experiences influence sports consumption both directly and indirectly, with the indirect effect predominating. Although immersion has a direct impact on consumption tendencies, its indirect pathway through team identification exhibits greater explanatory strength. This indicates that immersion alone seldom generates stable behavioral responses; its behavioral significance is realized primarily through the mediating role of identification.
(4) Motivational regulation moderates the structural pathways. Intrinsically regulated individuals rely more heavily on the indirect immersion → identification → consumption chain, whereas extrinsically regulated individuals respond more directly to immersive stimuli. This distinction underscores both the dependence of behavior on motivational orientation and the structural differentiation embedded in the behavioral-generation process.

### Recommendations

(1) Optimize immersive event design to strengthen emotional absorption and perceptual focus
  Event organizers should systematically enhance audience immersion by integrating visual intensity, contextual pacing, and interactive mechanisms that generate a strong sense of presence and attenuated time perception. Specific strategies include synchronizing lighting and sound, choreographing dramatic progressions, and delivering immersive commentary to sustain attentional concentration and intensify emotional resonance.
(2) Develop coherent, relatable team symbol systems to activate identification mechanisms
  Sports organizations should advance the storytelling, personification, and ritualization of team symbols. By standardizing visual elements, reinforcing player personas, and cultivating fan participation rituals, they can foster stronger psychological belonging and social identification. Such practices shift event consumption from passive viewing to active identification, thereby deepening audience attachment.
(3) Implement segmented positioning strategies aligned with distinct motivational structuresGiven variations in motivational regulation, targeted approaches are required: For intrinsically regulated audiences, prioritize community integration, cultural identity, and opportunities for identity construction. For extrinsically regulated audiences, emphasize real-time feedback, incentive-driven stimuli, and accessible interactive experiences. In practice, motivational assessments and behavioral-tracking tools should be employed to align communication and engagement strategies with the psychological profiles of different groups.
(4) Embed identity-construction mechanisms across communication and operations
  Identity construction should be treated as a foundational psychological module within event communication, fan engagement, and product design. By systematically embedding identity labels, community affiliation, and value propositions, organizers can build an integrated psychological chain of immersion–identification–consumption. This approach positions audiences not merely as attention resources but as participants in enduring processes of identity formation and social belonging.

### Research limitations and future directions

Although this study makes meaningful contributions at both theoretical and empirical levels, several limitations should be acknowledged. The sample consisted predominantly of young adults aged 18–35, which restricts representativeness and raises concerns regarding the generalizability of the findings across older cohorts, diverse cultural contexts, and heterogeneous media environments. Moreover, the reliance on self-reported questionnaires within a cross-sectional design constrains the ability to establish causal relationships and capture temporal dynamics; longitudinal tracking and experimental approaches are therefore required to verify the robustness of the proposed mechanisms and to strengthen causal inference. In addition, the dependent variables focused primarily on consumption intentions rather than actual transactions or long-term engagement behaviors, which limits the behavioral extrapolation of the conclusions. Future studies should incorporate transaction records, social-media interactions, and multimodal behavioral indicators to enhance ecological validity and improve the precision of behavioral predictions.

## Data Availability

The original contributions presented in the study are included in the article/Supplementary Material, further inquiries can be directed to the corresponding author.

## References

[1] Yoshida M Gordon BS Nakazawa M Biscaia R. Conceptualization and measurement of fan engagement: empirical evidence from a professional sport context. J Sport Manag. (2014) 28(4):399–417. 10.1123/jsm.2013-0199

[2] Gao F Ma Y Qiao F Yan P Niu Z. The impact of sports event platforms on user experience an empirical analysis of tencent sports events. Sci Rep. (2024) 14(1):1–15. 10.1038/s41598-024-78524-x 39622874 PMC11612232

[3] Pine BJ Gilmore JH. The Experience Economy: Work is Theatre & Every Business a Stage. Boston, MA: Harvard Business School Press (1999).

[4] Kim J Ko E. The impact of virtual reality (VR) technology on sport spectators’ flow experience and satisfaction. Comput Hum Behav. (2019) 93:346–56. 10.1016/j.chb.2018.12.040

[5] Ryan RM Deci EL. Self-determination theory and the facilitation of intrinsic motivation, social development, and well-being. Am Psychol. (2000) 55(1):68–78. 10.1037/0003-066X.55.1.68 11392867

[6] Vallerand RJ Rousseau FL. Intrinsic and extrinsic motivation in sport and exercise. In: Singer RN Hausenblas HA Janelle CM, editors. Handbook of Sport Psychology, 2nd ed., New York, NY: Wiley (2001). p. 389–416.

[7] Pelletier LG Rocchi MA Vallerand RJ Deci EL Ryan RM. Validation of the revised sport motivation scale (SMS-II). Psychol Sport Exerc. (2013) 14(3):329–41. 10.1016/j.psychsport.2012.12.002

[8] Deci EL Ryan RM. Intrinsic Motivation and Self-determination in Human Behavior. New York, NY: Springer (1985).

[9] Gagné M Deci EL. Self-determination theory and work motivation. J Organ Behav. (2005) 26(4):331–62. 10.1002/job.322

[10] Vallerand RJ. A hierarchical model of intrinsic and extrinsic motivation for sport and physical activity. In: Hagger MS Chatzisarantis NLD, editors. Intrinsic Motivation and Self-Determination in Exercise and Sport. Champaign, IL: Human Kinetics (2007). p. 255–79.

[11] Mastromartino B Zhang JJ. Affective outcomes of membership in a sport fan community. Front Psychol. (2020) 11:881. 10.3389/fpsyg.2020.00881 32457685 PMC7225319

[12] Tajfel H Turner JC. The social identity theory of intergroup behavior. In: Worchel S Austin WG, editors. Psychology of Intergroup Relations. Chicago, IL: Nelson-Hall (1986). p. 7–24.

[13] Lock D Heere B. Identity crisis: a theoretical analysis of “team identification” research. Eur Sport Manag Q. (2017) 17(4):413–35. 10.1080/16184742.2017.1306872

[14] Madrigal R. Measuring the multidimensional nature of sporting event performance consumption. J Leis Res. (2006) 38(3):267–92. 10.1080/00222216.2006.11950079

[15] Jackson SA Marsh HW. Development and validation of a scale to measure optimal experience: the flow state scale. J Sport Exerc Psychol. (1996) 18(1):17–35. 10.1123/jsep.18.1.17

[16] Chalip L Green BC Hill B. Effects of sport event media on destination image and intention to visit. J Sport Manag. (2003) 17(3):214–34. 10.1123/jsm.17.3.214

[17] Lock D Funk DC Doyle JP. Examining the longitudinal structure, stability, and dimensional interrelationships of team identification. J Sport Manag. (2014) 28(2):119–35. 10.1123/jsm.2012-0191

[18] Trail GT Anderson DF Fink JS. Sport spectator consumption behavior. Sport Mark Q. (2003) 12(1):8–17. 10.1177/106169340301200102

[19] Klein M. Review of the book Sociological Theory in the Digital Age, by Gabe Ignatow & Laura Robinson. Sociol čas. / Czech Sociol. Rev. (2019) 55(3):412–3. Available online at: https://sreview.soc.cas.cz

[20] Csikszentmihalyi M. Flow: The Psychology of Optimal Experience. New York, NY: Harper & Row (1990).

[21] Lock D Funk DC. The multiple in-group identity framework. Sport Manag Rev. (2016) 19(2):85–96. 10.1016/j.smr.2015.10.001

[22] Theodorakis ND Kaplanidou K Karabaxoglou I. Effect of event service quality and satisfaction on happiness among runners of a recurring sport event. Leis Sci. (2015) 37(1):87–107. 10.1080/01490400.2014.938846

[23] Heere B James JD. Sports teams and their communities: examining the influence of external group identities on team identity. J Sport Manag. (2007) 21(3):319–37. 10.1123/jsm.21.3.319

[24] Chirkov V Ryan RM Kim Y Kaplan U. Differentiating autonomy from individualism and independence: a self-determination theory perspective on internalization of cultural orientations and well-being. J Pers Soc Psychol. (2003) 84(1):97–110. 10.1037/0022-3514.84.1.97 12518973

[25] Putnick DL Bornstein MH. Measurement invariance conventions and reporting: the state of the art and future directions for psychological research. Dev Rev. (2016) 41:71–90. 10.1016/j.dr.2016.06.004 27942093 PMC5145197

[26] Sass DA Schmitt TA. Testing measurement and structural invariance. In: Teo T, editor. Handbook of Quantitative Methods for Educational Research. Rotterdam: SensePublishers (2013). p. 315–45.

